# Different Harmonic Characteristics Were Found at Each Location on TCM Radial Pulse Diagnosis by Spectrum Analysis

**DOI:** 10.1155/2018/9018271

**Published:** 2018-07-05

**Authors:** Yun-Ning Tsai, Yi-Chia Huang, Sunny Jui-Shan Lin, Shen-Ming Lee, Yung-Yen Cheng, Yu-Hsin Chang, Yi-Chang Su

**Affiliations:** ^1^Graduate Institute of Chinese Medicine, College of Chinese Medicine, China Medical University, Taichung 40402, Taiwan; ^2^Chander Clinic, Taipei 10646, Taiwan; ^3^Department of Chinese Medicine, Tri-Service General Hospital, National Defense Medical Center, Taipei 11490, Taiwan; ^4^National Research Institute of Chinese Medicine, Ministry of Health and Welfare, Taipei 11221, Taiwan; ^5^Department of Statistics, Feng-Chia University, Taichung 40764, Taiwan; ^6^Center for Survey Research, Research Center for Humanities and Social Sciences, Academia Sinica, Taipei 11529, Taiwan; ^7^The Department of Internal Medicine, Nantou Hospital of the Ministry of Health and Welfare, Nantou 54062, Taiwan; ^8^Taichung Chander Clinic, Taichung 40356, Taiwan; ^9^School of Chinese Medicine, College of Chinese Medicine, China Medical University, Taichung 40402, Taiwan

## Abstract

**Purpose:**

This study aimed to clarify whether it is appropriate to choose any measurement location for pulse diagnosis research.

**Methods:**

A total of 37 subjects were recruited and measured for pulse pressure waves at 18 locations (9 per hand of “three positions and nine indicators”). These data were Fourier-transformed to the frequency spectrum, and the harmonics of C0-C10 of each location were obtained. Box plots of the harmonics were generated using SPSS v.22.0 and R v.3.4.1. Data were compared with multivariate analysis of variance (MANOVA) with a randomized block design.

**Results:**

The results showed that certain harmonics were different at different positions and different indicators; the harmonics of the same indicator at different positions (except for C8 and C10) and those of different indicators for the same position (except for C4 and C5) were significantly different (p<0.05).

**Conclusions:**

In future researches of pulse diagnosis, due to the significant differences between positions and indicators, it is recommended that the measurement position should be carefully chosen instead of choosing any measurement location to ensure the integrity of the acquired information for further analyzing physiological or pathological status.

## 1. Introduction

**Figure 1:**
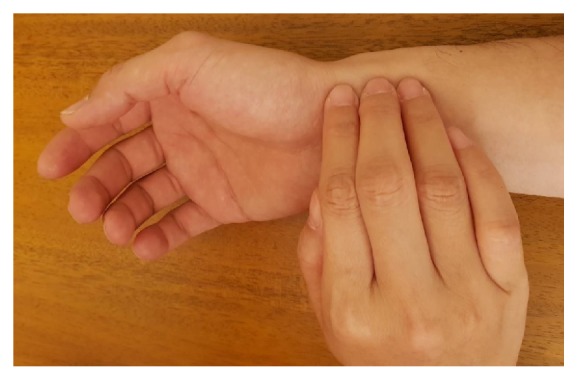
Pulse diagnosis in clinical situation.

The “pulse diagnosis” of traditional Chinese medicine (TCM) refers to the physician's use of three fingers (index finger, middle finger, and ring finger) on three “positions”, i.e., inch (寸), bar (關), and cubit (尺) of the patient to perceive the pulse by pressing on three “indicators”, i.e., superficial (浮), medium (中), and deep (沈), to observe the states of different organs [1, 2]. This method is derived from the positioning method of “three positions and nine indicators” and has been widely used in clinical field [1]. The clinical situation of pulse diagnosis of TCM is shown in Figure 1.

Different measurement locations of pulse were chosen by different researches, such as (i) single location, e.g., inch (without mentioning left inch or right inch) [3], left inch [4], right bar [5], or bar (without mentioning left bar or right bar) [6]; (ii) three locations (inch, bar, and cubit) [7], or (iii) the pulse ridge [8]. Compared with time-domain analysis [3, 9, 10], spectrum analysis can reveal additional physiological and pathological messages [6, 11–20] in pulse diagnosis analysis.

On the other hand, according to the “resonance theory” proposed by Wang et al. that the pulse wave resonates with the heartbeat and the pulse wave can also be broken into a number of harmonics that match the natural frequency of the resonance [21], these harmonics may also correspond to different organs. Combining resonance theory and pulse spectrum analysis, harmonics analysis has been widely used in pulse diagnosis researches of TCM in recent years [1, 2, 22, 23].

We wondered “Is it appropriate to choose any measurement location in pulse diagnosis researches?” In this study, we used harmonics analysis to investigate this question.

## 2. Materials and Methods

### 2.1. The Breakdown of Pulse Waves into Harmonics and Their Meanings

In the arterial system, the pulse generates periodic pulse waves with the heartbeat. Wang et al. dissertated that the matching condition formed by the driving force generated by the heart and the natural frequency of the resonance generated by the heartbeat and the arterial system determined the change in the pulse waves [2, 24–27]. Wang et al. also found that, in the entire arterial system, a pulse wave can be broken down into a plurality of distributed stationary waves that are associated with the harmonic components of the heartbeat-derived periodic force [2]. When the intrinsic frequencies of these waves are integer multiples, the efficiency of their power transmission gives rise to the best match with the whole arterial system [2]. Based on the mathematical theory of Fourier series, the original pulse wave can be broken down into a number of sine waves with a frequency that is an integer multiple of the frequency of the heartbeat, and these sine waves are defined as harmonics [28].

### 2.2. Recruitment of Test Subjects

The study was conducted from July 2011 to October 2012, in the Department of Cardiology of the Medical Center of Tri-Service General Hospital of Taiwan, as a subproject under the project “Pulse Spectrum Analysis to Aid Diagnosis of Coronary Artery Disease”. The inclusion criteria for the subjects were as follows: at least 18 years of age, either sex, seeking treatment on chest pain or angina, and required hospitalization for further examinations. The exclusion criteria included acute myocardial infarction, arrhythmia, heart valve disease, cancer, severe infection, pregnancy, severe mental illness, and a radial artery pulse that could not be measured. The content of the research project was approved by the Institutional Review Board (IRB) (Case #: TSGHIRB 100-05-016).

### 2.3. Instrument

**Figure 2:**
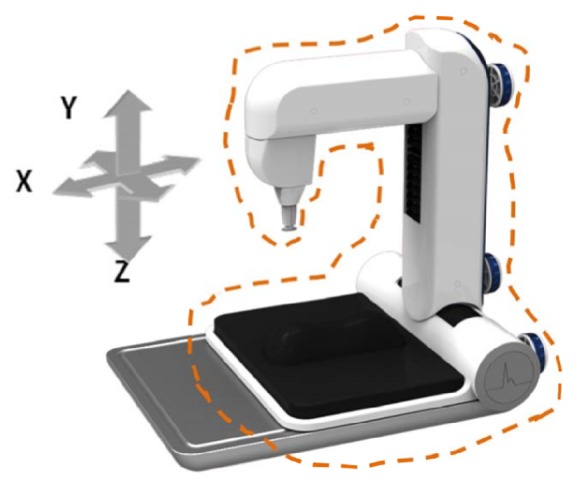
The mechanism of this instrument is the positioning mechanism of X-axis, Y-axis, and Z-axis adjustments and lateral structure movements. The dotted block can be moved axially to align with the front of the base.

**Figure 3:**
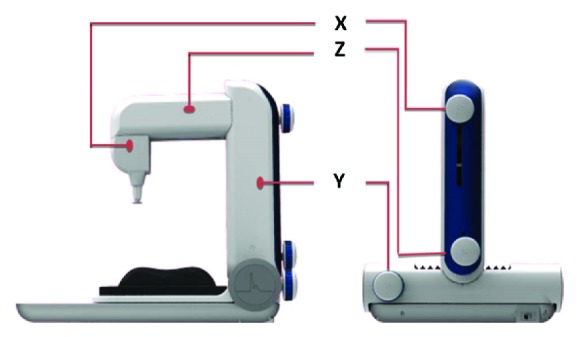
Each knob and action position of X-, Y-, and Z-axes are indicated by the orange lines.

**Figure 4:**
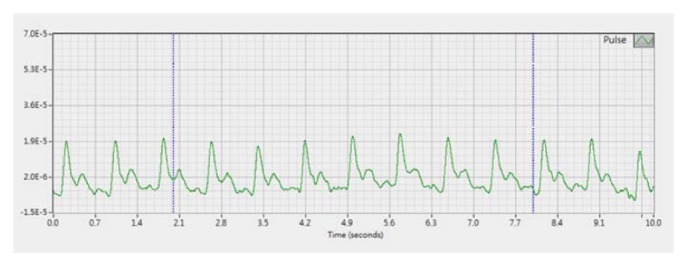
A typical arterial pulse waveform obtained from PDS-2010 Skylark Pulse Analysis System.

A PDS-2010 Skylark Pulse Analysis System (developed by Cologne Technology Instruments Co., Ltd., Taiwan, the Ministry of Health and Welfare, medical equipment license number: 003627) was used in this study [29]. The specifications of the measuring host of this instrument are described as follows: dimension: 341.5H *∗* 269W *∗* 375L (mm); weight: 4.7 KG; temperature and humidity percentage of keep and transportation: 5°C~ 40°C and 30~80 %; operating temperature: 20°C~40°C; sensor: input and output resistance 350 Ohm. The photo of the instrument is shown in Figures 2 and 3. By measuring the horizontal and vertical changes on the X-, Y-, and Z-axes of the measurement unit, the pulse waves in different positions and different indicators were determined based on the analogy electrical signals of wrist artery waves; subsequently, the analogous electrical signals were converted into digital data and stored on a computer. A typical arterial pulse waveform obtained by this instrument is shown in Figure 4.

### 2.4. Operational Definition of “Three Positions and Nine Indicators”

**Figure 5:**
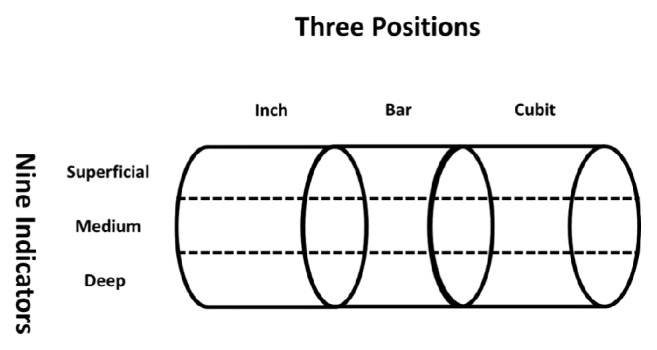
*Schematic diagram of “three positions and nine indicators”*. The abbreviations for the “three positions and nine indicators” are as follows: SRI=superficial indicator within the right inch (*右寸浮*); MRI=medium indicator within the right inch (*右寸*中); DRI=deep indicator within the right inch (*右寸沈*); SRB=superficial indicator within the right bar (*右關浮*); MRB=medium indicator within the right bar (*右關*中); DRB=deep indicator within the right bar (*右關沈*); SRC=superficial indicator within the right cubit (*右尺浮*); MRC=medium indicator within the right cubit (*右尺*中); DRC=deep indicator within the right cubit (*右尺沈*); SLI=superficial indicator within the left inch (*左寸浮*); MLI=medium indicator within the left inch (*左寸*中); DLI=deep indicator within the left inch (*左寸沈*); SLB=superficial indicator within the left bar (*左關浮*); MLB=medium indicator within the left bar (*左關*中); DLB=deep indicator within the left bar (*左關沈*); SLC=superficial indicator within the left cubit (*左尺浮*); MLC=medium indicator within the left cubit (*左尺*中); and DLC=deep indicator within the left cubit (*左尺沈*).

The method of “three positions and nine indicators” in TCM refers to the fact that at the pulsating site of the wrist radial artery, in the direction from the wrist to the elbow, the wrist area is divided into three positions, i.e., inch (寸), bar (關), and cubit (尺), while each position is divided into three indicators, i.e., superficial (浮), medium (中), and deep (沈), in the vertical direction, giving rise to a total of nine indicators, called the “three positions and nine indicators” [30, 31]. With both hands, there are a total of 18 locations. We proposed operational definitions for the three positions inch, bar, and cubit [32]. For the operational definitions of the three indicators, superficial, medium, and deep, pressures in three weight ranges, 0-50 g, 50-90 g, and 90-120 g, were exerted downward by the instrument probe, and the depth that returned the most distinct pulse wave was assigned to each of the indicators (in Figure 5, the abbreviations of “three positions and nine indicators” are included).

### 2.5. Research Procedure

**Figure 6:**
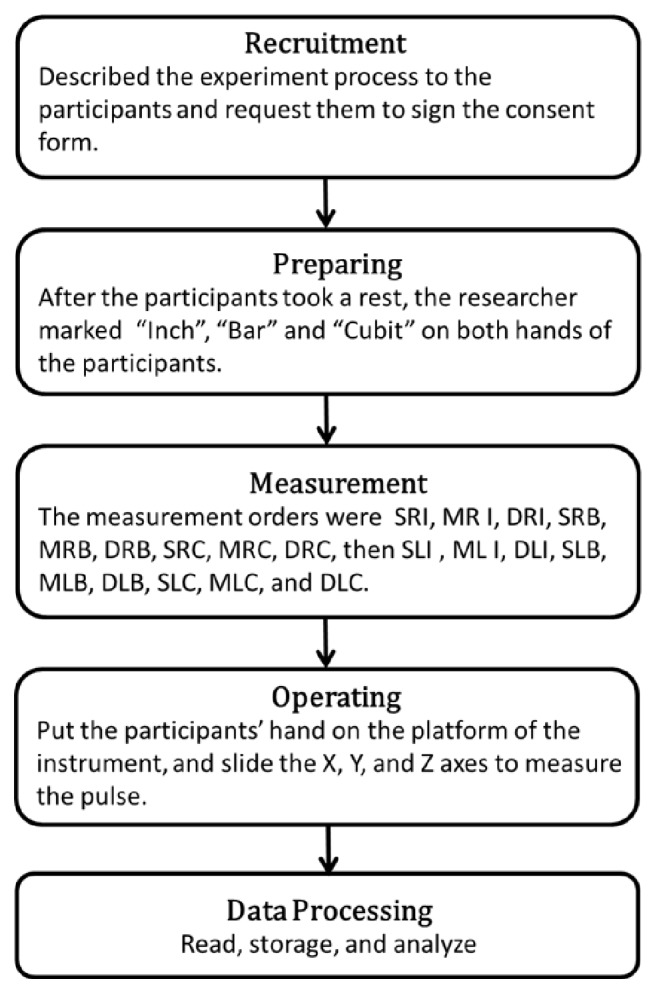
The flow chart.

**Figure 7:**
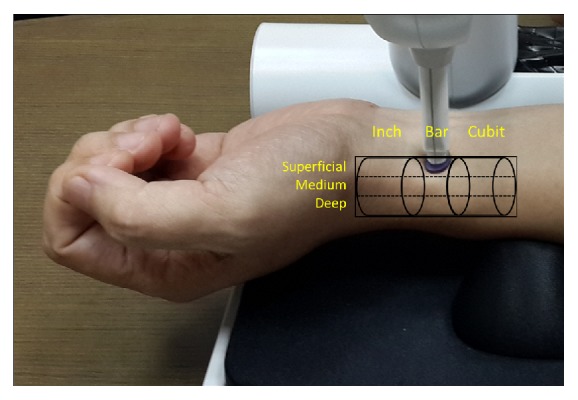
The photo of experiment.

All of the procedures were conducted in a bright and quiet room, and the room temperature was kept between 25°C and 26°C. A well-trained researcher conducted all of the procedures using the same instrument to avoid the measurement error. The flow chart and photo of experiment are shown in Figures 6 and 7.

### 2.6. Data Processing

The pulse wave data were subjected to fast Fourier transformation (FFT) using LabVIEW 7 software (National Instruments Co., USA) to generate the pulse wave spectral harmonics for each location. The value of each harmonic was defined as follows: Ao is the direct current (DC) portion of the pulse wave spectrum; An is the amplitude of the nth harmonic of the spectrum; and Cn is the percentage of the nth harmonic in the DC portion [28], i.e., Cn = (An / Ao) × 100%. When n = 0, Co = Ao. In the present study, n was set to 0–10 [33, 34]. After data processing, C0-C10 at each location was obtained.

### 2.7. Statistical Analysis and Method

Two statistical analysis software packages, SPSS v.22.0 and R v.3.4.1, were used for analyzing the data. The basic information of the subjects is presented in the form of descriptive statistics. First, box plots [35, 36] were generated to observe the distribution of each of the harmonics at the 18 locations. Then, because “three positions and nine indicators” covers the plane variables inch, bar, and cubit, i.e., the positions, as well as the depth variables, superficial, medium, and deep, i.e., the indicators, and the different subjects were expected to exhibit variations that would impact the test results, a randomized block design was implemented to facilitate the multivariate variance analysis (MANOVA) [37, 38]. In the MANOVA, different subjects were regarded as blocks that were viewed as independent variables, while different positions (inch, bar, and cubit on both hands, for a total of six positions) and different indicators (superficial, medium, and deep, at a total of three positions) were also viewed as independent variables, so that the parameters of the spectrum analysis could be compared between different subject blocks, different positions, or different indicators. p<0.05 was deemed statistically significant.

## 3. Results

### 3.1. Basic Information

A total of 37 subjects who met the inclusion criteria were included in the study. They all underwent measurements of the pulse waves of the three positions and nine indicators on both hands. The age, height, weight, and BMI of the subjects were 59.54 ± 10.22 (36.25-78.25) years, 166.59 ± 8.62 (150-194) cm, 71.96 ± 10.87 (54-100) kg, and 25.91 ± 3.24 (20.19-32.66) kg/m^2^, respectively.

### 3.2. The C0-C10 of the 18 Locations of the Two Hands: Box Plots

**Figure 8:**
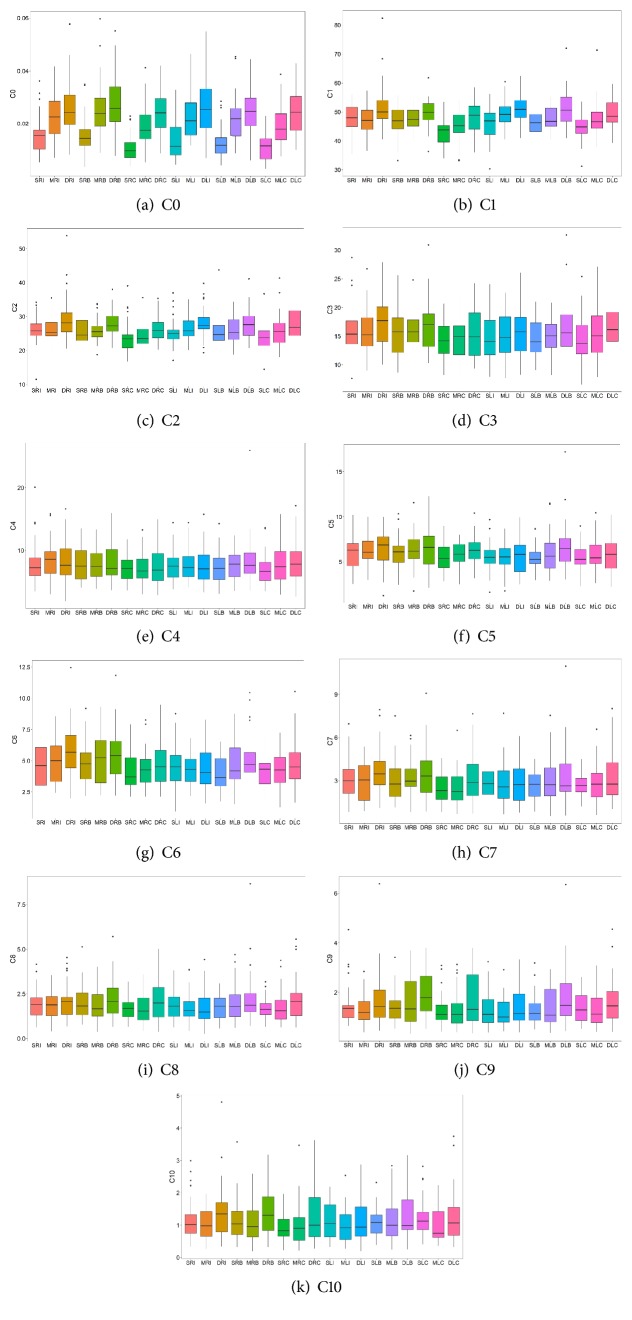
*The C0-C10 of the 18 locations on both hands, presented as box plots*. X-axis: abbreviations of the 18 locations (refer to the description of Figure 5). (a) The Y-axis represents the amplitude of the pulse wave in the direct current portion, i.e., C0. (b)-(k) The Y-axis represents the percentage of the amplitude of the harmonics of C1-C10 relative to C0. Each box represents the 50% range of variation of the measurements of 37 subjects at the location, and the horizontal line on the box represents the median of the measurements of 37 subjects at the location. “.” means outliers.

To observe the distribution of harmonics at 18 locations, the box plots of C0, C1, C2,..., C10 are presented in Figures 8(a)–8(k). Taking Figure 8(a) as an example, the medians of C0 at the 18 locations varied, and the medians of C0 at the inch, bar, and cubit of both hands gradually increased with the increase in the depth of the superficial, medium, or deep indicator. For example, in the case of the right inch, the medians gradually increased as the depth increased (i.e., SRI, MRI, and DRI; the related abbreviations have been presented in Figure 5), and a similar pattern was present in the cases of the right bar, right cubit, left inch, left bar, and left cubit. In Figures 8(b) and 8(c), the medians of C1 and C2 at the 18 locations, except for the superficial and medium indicators of the right inch, and the medians of the deep indicator were significantly higher than those of the superficial indicator. In Figure 8(d), the medians of C3 at the 18 locations slightly decreased in the cases of the superficial and medium indicators of the right inch, but in the cases of the superficial and medium indicators of the right bar and the medium and deep indicators of the right cubit, the medians did not increase, while those of the right inch, right bar, or right cubit of C3 at the deep indicator were significantly higher than those at the superficial indicator. Moreover, the medians of C1, C2, and C3, in addition to the above-mentioned positions, increased with the depth of the indicator. In Figures 8(f) and 8(g), the medians of C5 and C6 at the 18 locations (except in the cases of C5 at the right inch and C6 at the left inch and left cubit) exhibited the same pattern. As shown in Figures 8(e), 8(h), 8(i), 8(j), and 8(k), the medians of C4, C7, C8, C9, and C10 at the 18 locations and their distributions exhibited slightly different patterns. These results indicate that even the amplitude of the same harmonic can be different at different locations.

### 3.3. The C0-C10 of the 18 Locations of the Two Hands: MANOVA Results

To further confirm whether the differences above were statistically significant, MANOVA was performed.  Table 1 shows whether the harmonics of different positions were the same. Taking C0 as an example, among the 37 subject blocks, the differences were significant (p<0.05); the means of C0 at the three indicators (i.e., superficial, medium, and cubit) were significantly different between the six positions (right inch, right bar, right cubit, left inch, left bar, and left cubit). The means of C8 and C10 at six positions showed no significant differences, but the other harmonics exhibited similar results to those of C0 regarding the subject blocks and six positions (p<0.05).

Table 2 shows whether the harmonics at different indicators were identical. Taking C0 as an example, the differences were significant between the 37 subject blocks; the means of C0 at the six positions (right inch, right bar, right cubit, left inch, left bar, and left cubit) were significantly different at the three depths (superficial, medium, and deep; p<0.05). For C1-C10, except C4 and C5 at the superficial, medium, and deep indicators at the right and left inches, bars, and cubits, which were not significantly different, the harmonics exhibited similar results to those of C0 regarding the subject blocks and six positions (p<0.05).

## 4. Discussion

Pulse diagnosis at the wrist radial artery in TCM uses the pulse beat status that is generated by vascular pressure fluctuations at different locations to assess the physiological and pathological states of various organs [1, 30]. The pulsation condition depends on the matching condition formed by the driving force generated by the heart and the natural frequency of the resonance of the heartbeat and arterial system [2]. Regarding the acquisition of pulse waves from the wrist radial artery, pulse diagnosis studies have not yet investigated whether the information is affected by the differences resulting from different states due to pulsations at different locations. Therefore, the measurement location for pulse diagnosis varied and coexisted. In this regard, this study represents the attempt in clarifying the issue with experimental data to fill the gap.

From the perspective of medical engineering, Wang et al. proposed the organ-artery coupled resonance theory [24], which argues that each harmonic that is derived from the breakdown of the pulse wave obtained by measurement at individual locations can correspond to the resonance condition of different organs in the heart-artery system [1, 2]. On the other hand, according to the idea of “three positions and nine indicators” of the TCM pulse diagnosis, different locations correspond to different organs [30]. In spectrum analysis, if using the pulse wave that is acquired from any one or three individual locations for diagnosis, it is not in agreement with the idea of pulse diagnosis positioning in TCM. As shown in Figure 8, even the mean of the same harmonic at a certain location was different from that at each of the other locations, and then if all the harmonics were integrated, the harmonics at different locations were not identical. This finding indicates that the practice of pulse diagnosis at different locations in TCM is by no means meaningless.

The harmonics at different positions, except for C8 and C10, were significantly different within different indicators (Table 1). Within different indicators, except for C4 and C5, the harmonics were significantly different at different positions (Table 2). The results indicated that, in TCM, the frequency features of the pulse waves of the different positions and indicators are indeed different. These findings also have implications on the choice of the measurement locations in pulse diagnosis spectral studies. It is strongly necessary to put forward the theoretical basis for choosing either inch, bar, cubit [3, 5, 7], pulse ridge [8], or deeper location [39]. Stronger theoretical evidence is needed for the chosen location to avoid missing the pulse wave information that is implied at other locations.

Various methods were chosen on pulse wave analyses, such as the pulse ridge (the highest point of the pulse wave) [8], the maximum pulse pressure [39], and the largest pulse amplitude [40], to perform the measurement. In terms of spectrum harmonic analysis, because the pulse ridge is located at a shallower location of the pulse wave and the harmonics at different depths were different (Table 2), it is impossible for the harmonics of the pulse ridge to represent the harmonics at other locations. Further, the locations of the maximum pulse pressure and the largest pulse amplitude may shift on different planes and at different depths. Based on Tables 1 and 2, once the locations of the maximum pulse pressure and the largest pulse amplitude shift horizontally or vertically, the results of the harmonics will change with a change at the measurement location.

In fact, when choosing the measurement location in studies on pulse wave analysis, some investigators indeed applied the pulse diagnosis positioning theory of TCM. For example, Huang et al. applied the theory that the left inch reflects the heart to analyze the spectral harmonic energy ratio of each harmonic of the patients with palpitation [4]. Chao et al. applied the theory that the right bar reflects the digestion function to observe the change in the pulse wave of the right bar caused by the hot-attribute aged ginger tea [5]. The methods used in those studies indicated that different pulse diagnosis locations exhibit the organ-corresponding characteristics or a stronger connection to different organs. Therefore, we should be more cautious in choosing the measurement location in harmonic analysis and the interpretation of the state of a specific organ using the pulse wave that is measured at different locations. The results of this study indicate that the values of the harmonics on the plane of different positions and at different depths varied, suggesting that the harmonic of a certain location is different from that at another location. How to select the organ-corresponding location to analyze the spectral features of physiological or pathological conditions requires more in-depth investigations.

Liao et al. investigated the harmonics of the right inch, right bar, right cubit, left inch, left bar, and left cubit of pregnant women [7] and found that the harmonic at cubit was unique in the stage of pregnancy, but they did not clearly describe the depths of the actual measurement of the six positions. In this study, we showed that the harmonics of the six positions themselves varied and that the harmonics also varied at different depths, which again indicates that when choosing the measurement location in a study of pulse wave spectral analysis, more complete consensus and more rigorous design (e.g., simultaneously taking different positions and different indicators into account) are needed.

Wang et al. showed that the shapes of the pulse waves detected in the locations of the inch, bar, and cubit of the same hand were almost identical, whereas the shapes of the pulse waves of the left and right hands were not the same. In addition, Wang et al. found the differences in the pulse waves measured at the superficial, medium, and deep indicators could not be noticed with the naked eye [1]. However, such findings lack corroboration or support from experimental data. In this study, with rather complete research data, we present some novel answers regarding the pulse waves in the locations of the inch, bar, and cubit and at the superficial, medium, and deep indicators in the spectrum analysis.

The reason we did not set up a control group is because our goal is to clarify whether the harmonic characteristics at 18 locations are consistent among all people to provide objective advice on the measurement locations for future pulse diagnosis researches. Since the results of this study showed that the harmonics obtained at 18 locations were actually not the same in the population with the disease, the main question of this study has been answered. Based on this result, we did not set up a control group to continue to verify whether the harmonic characteristics at 18 locations in the control group were the same or not.

The major contribution of this study is not to indicate which measurement location is correct but to first confirm whether the harmonics at a single location are the same as those at other locations. Our focus is that if the harmonics are the same at each location, the measurement location could be replaced with each other. However, if it is not feasible, we must carefully consider the measurement location (for example, return to “three positions and nine indicators” in TCM or further explore other suitable location which could represent each location, which could not be answered in our study yet). This is really a long-term neglected preparatory work in the past research on pulse wave analysis. Also, due to the bottleneck, since pulse wave harmonic analysis is applied to the analysis of pulse signals for decades, the results of these studies are also difficult to integrate.

## 5. Conclusions

The harmonic characteristics of the pulse waves presented by the radial artery in the wrist at different positions and different indicators were not identical. It recommended that it is not appropriate to choose any measurement location in future studies on the spectrum analysis of pulse diagnosis. Besides, careful consideration about the measurement locations should be made to ensure the completeness and reliability of the information to be analyzed for further physiological or pathological status.

## Figures and Tables

**Table 1:** MANOVA results for whether the plane vectors of the C0-C10 of three indicators at the six positions of the right inch, right bar, right cubit, left inch, left bar, and left cubit were identical.

** Harmonic**	**Variable**	**DOF**	**Wilks's lambda**	**Approx F**	**DOF of numerator**	**DOF of denominator**	**p-value**
C0	Subject block	36	0.32330	3.7725	72	358	< 2.2e-16*∗*
	Superficial, Medium, Deep	5	0.79450	4.3638	10	358	8.720e-06*∗*

C1	Subject block	36	0.36219	3.2897	72	358	8.883e-14*∗*
	Superficial, Medium, Deep	5	0.77764	4.7970	10	358	1.757e-06*∗*

C2	Subject block	36	0.26344	4.7153	72	358	<2.2e-16*∗*
	Superficial, Medium, Deep	5	0.85627	2.8882	10	358	0.001751*∗*

C3	Subject block	36	0.18008	6.7450	72	358	<2e-16*∗*
	Superficial, Medium, Deep	5	0.86304	2.7360	10	358	0.00295*∗*

C4	Subject block	36	0.12741	8.9578	72	358	<2.2e-16*∗*
	Superficial, Medium, Deep	5	0.87549	2.4610	10	358	0.007431*∗*

C5	Subject block	36	0.24301	5.1142	72	358	<2.2e-16*∗*
	Superficial, Medium, Deep	5	0.89588	2.0232	10	358	0.03022*∗*

C6	Subject block	36	0.20725	5.9497	72	358	<2.2e-16*∗*
	Superficial, Medium, Deep	5	0.82734	3.5586	10	358	0.0001642*∗*

C7	Subject block	36	0.22887	5.4210	72	358	<2.2e-16*∗*
	Superficial, Medium, Deep	5	0.88574	2.2391	10	358	0.01531*∗*

C8	Subject block	36	0.30299	4.0608	72	358	< 2.2e-16*∗*
	Superficial, Medium, Deep	5	0.92089	1.5059	10	358	0.1352

C9	Subject block	36	0.24378	5.0984	72	358	< 2.2e-16*∗*
	Superficial, Medium, Deep	5	0.90030	1.9302	10	358	0.04016*∗*

C10	Subject block	36	0.28169	4.3962	72	358	< 2.2e-16*∗*
	Superficial, Medium, Deep	5	0.90761	1.7781	10	358	0.06313

The means of the C0-C10 of the 37 subjects, in blocks of individual subjects, were tested, whether they were identical at the six positions (right inch, right bar, right cubit, left inch, left bar, and left cubit). DOF means degrees of freedom. Wilks's lambda represents the ratio of the intragroup square sum over the total square sum, and Approx F represents the F-statistic of Wilks's lambda after the transformation; *∗* denotes p<0.05.

**Table 2:** MANOVA results for whether the depth vectors of the C0-C10 at the six positions on the three indicators were identical.

**Harmonic**	**Variable**	**DOF**	**Wilks's lambda**	**Approx F**	**DOF of numerator**	**DOF of denominator**	**p-value**
C0	Subject block	36	0.05672	6.3086	72	142	< 2.2e-16*∗*
	Right & left (Inch, Bar, Cubit)	2	0.33023	26.2760	4	142	2.583e-16*∗*

C1	Subject block	36	0.18679	2.5910	72	142	63928e-07*∗*
	Right & left (Inch, Bar, Cubit)	2	0.70390	6.8130	4	142	4.799e-05*∗*

C2	Subject block	36	0.12444	3.6185	72	142	3.202e-11*∗*
	Right & left (Inch, Bar, Cubit)	2	0.64390	8.7405	4	142	2.454e-06*∗*

C3	Subject block	36	0.06105	6.0100	72	142	< 2.2e-16*∗*
	Right & left (Inch, Bar, Cubit)	2	0.77240	4.8930	4	142	0.001002*∗*

C4	Subject block	36	0.03648	8.3535	72	142	< 2.2e-16*∗*
	Right & left (Inch, Bar, Cubit)	2	0.94678	0.9840	4	142	0.4184

C5	Subject block	36	0.06926	5.5216	72	142	< 2.2e-16*∗*
	Right & left (Inch, Bar, Cubit)	2	0.90371	1.8433	4	142	0.1238

C6	Subject block	36	0.07365	5.2949	72	142	< 2.2e-16*∗*
	Right & left (Inch, Bar, Cubit)	2	0.72075	6.3155	4	142	0.00001048*∗*

C7	Subject block	36	0.11230	3.9130	72	142	2.092e-12*∗*
	Right & left (Inch, Bar, Cubit)	2	0.83201	3.4193	4	142	0.01057*∗*

C8	Subject block	36	0.12290	3.6535	72	142	2.308e-11*∗*
	Right & left (Inch, Bar, Cubit)	2	0.85895	2.8041	4	142	0.02806*∗*

C9	Subject block	36	0.14050	3.2894	72	142	7.317e-10*∗*
	Right & left (Inch, Bar, Cubit)	2	0.81966	3.7113	4	142	0.006632*∗*

C10	Subject block	36	0.12595	3.5850	72	142	4.39e-11*∗*
	Right & left (Inch, Bar, Cubit)	2	0.82558	3.5706	4	142	0.008303*∗*

The means of the C0-C10 of the 37 subjects, in blocks of individual subjects, were tested, whether the means of the harmonics of the six positions (right inch, right bar, right cubit, left inch, left bar, and left cubit) were identical on the three indicators (superficial, medium, and deep). DOF means degrees of freedom. Wilks's lambda represents the ratio of the intragroup square sum over the total square sum, and Approx F represents the F-statistic of Wilks's lambda after the transformation; *∗* denotes p<0.05.

## Data Availability

The data sets generated and analyzed during the current study are available from the corresponding author on reasonable request.
